# Successful Anesthetic Management for Laparoscopic Gynecological Surgery in Adult-Onset Alexander’s Disease: A Case Report

**DOI:** 10.7759/cureus.83287

**Published:** 2025-05-01

**Authors:** Christian Maldonado-Crespo, Julián J Zayas-Vélez, Elfren Colón-Rodríguez, Maria J Crespo

**Affiliations:** 1 Anesthesiology, University of Puerto Rico, School of Medicine, San Juan, PRI; 2 Physiology and Anesthesiology, University of Puerto Rico, School of Medicine, San Juan, PRI

**Keywords:** alexander's disease, general anesthesia, gynecologic surgical procedures, neurodegenerative diseases, perioperative care

## Abstract

Alexander's disease is a rare and progressive neurodegenerative disorder that presents significant challenges for anesthetic management due to its effects on the central nervous system. The disease stems from mutations in the glial fibrillary acidic protein (i.e., GFAP) gene, leading to impaired myelination and neurological complications such as bulbar dysfunction, spasticity, and autonomic instability. This case report presents the anesthetic management of a 34-year-old female patient diagnosed with Alexander's disease type II (i.e., adult-onset), cerebellar ataxia, and dysarthria. She underwent bilateral laparoscopic tubal sterilization and intrauterine device removal. After a thorough evaluation of the anesthetic options and consideration of the neurological complications associated with her condition, the procedure was successfully performed without complications under general anesthesia. To the best of our knowledge, this is the first documented case of a patient with Alexander’s disease type II undergoing a gynecological procedure under general anesthesia. This case highlights the complex considerations necessary in anesthetic care for patients with Alexander’s disease type II, including aspiration risk from bulbar dysfunction, altered response to neuromuscular blocking agents due to spasticity, and the potential for autonomic instability during surgical stimulation. Furthermore, it emphasizes the importance of a tailored anesthetic plan combined with vigilant perioperative monitoring in the management of these patients.

## Introduction

Alexander's disease is part of a group of neurogenetic diseases known as leukodystrophies that are attributed to mutations of the glial fibrillary acidic protein, or GFAP, gene. This condition affects the white matter of the central nervous system with or without peripheral nervous system (PNS) involvement and is characterized by abnormal development or destruction of the myelin sheath [1]. It is a rare neurodegenerative disease with an incidence of 1 case per 2.7 million people and represents 1.6% of all leukodystrophies [1]. This disease consists of various subtypes according to onset and severity. Type II, also known as the adult form, is less severe, has a later onset than the other subtypes, and has a median survival age of 25 years [1]. Symptoms can vary and may include progressive ataxia, which affects 75% of patients, and pseudobulbar and bulbar signs, which occur in approximately 50% of patients and may manifest in dysarthria/aphasia, dysphagia, or dysphonia [2]. Bulbar dysfunction poses challenges not only due to increased aspiration risk but also because of potential difficulties with airway reflexes and secretion management during extubation [1,2]. Furthermore, spasticity can affect positioning during surgery and may alter responses to neuromuscular blocking agents. In neurodegenerative conditions with bulbar or pseudobulbar involvement, the risk of aspiration is both insidious and progressive, mirroring the chronic microaspiration patterns seen in conditions like Parkinson’s disease. These risks often emerge later in the disease course as dysphagia and impaired oropharyngeal coordination worsen, compromising airway protection mechanisms. Although gastrointestinal dysmotility, such as gastroparesis, has not been specifically documented in Alexander’s disease, the presence of autonomic dysfunction raises suspicion for possible esophageal dysmotility or delayed gastric emptying, both of which may augment aspiration risk over time.

Additional clinical signs may include sleep disturbances (e.g., sleep apnea), postural alterations (e.g., scoliosis and kyphosis), palatal abnormalities, epilepsy, and diplopia. The treatment for Alexander's disease type II is supportive, focusing on managing these symptoms to improve quality of life. This treatment might involve therapies, such as anti-seizure medications for epilepsy, physical therapy for ataxia, and muscle-relaxant medications for spasticity. Some patients might require interventions like a ventriculoperitoneal shunt for hydrocephalus, or a gastrostomy tube for feeding difficulties [1]. The pre-existing symptomatology of altered mental status and frequent convulsions must be considered when tailoring anesthetic management.

We present the case of a 34-year-old patient with Alexander’s disease type II who successfully underwent intrauterine device (IUD) removal and laparoscopic bilateral tubal sterilization (BTS) under general anesthesia, utilizing midazolam, propofol, rocuronium, and fentanyl without complications.

## Case presentation

A 34-year-old woman weighing 50.8 kg, with a body mass index (BMI) of 21.87, gravida 1 para 1 (G1P1001), with a past medical history of beta-thalassemia trait, scoliosis, and cerebellar ataxia with dysarthria secondary to Alexander's disease type II, presented for a preoperative evaluation before her IUD removal and laparoscopic BTS. She reported mild bilateral weakness in her upper and lower extremities, which required her to use a walker and be assisted with dressing, standing up from the floor, and other activities. The patient did not experience headaches, seizures, dizziness, chest pain, or shortness of breath. Her gynecological history included menarche at age 14, with normal menstrual bleeding, and an uneventful spontaneous vaginal delivery at 40 weeks gestational age in 2009. She began experiencing progressive neurological symptoms in 2015 at age 25, including dysarthria, dysphagia, and gait instability. In 2017, she was given a presumptive diagnosis of adult-onset spinocerebellar ataxia (SCA) of an unknown subtype; however, genetic testing for common SCA mutations was negative, and the lack of a family history of neurodegenerative disease made the diagnosis uncertain. By 2021, her condition had worsened to include orthopnea, nocturnal urinary incontinence, and partial dependence on daily activities. This led to a referral for further neurological evaluation, during which Alexander’s disease was considered. A neurological exam in early 2022 revealed dysarthria, proximal lower limb weakness, bilateral dysmetria, hyperreflexia, and a positive Hoffman sign. Confirmatory genetic testing identified a pathogenic mutation in the glial fibrillary acidic protein (GFAP) gene, establishing a diagnosis of adult-onset Alexander’s disease [3].

The patient’s family history was notable in that the father had cerebrovascular accidents, and her mother had beta-thalassemia trait, arterial hypertension, type 2 diabetes mellitus, and hypothyroidism. Additionally, her daughter was also diagnosed with Alexander's disease. The patient worked as a clerk at the front desk of a hotel and did not smoke, use alcohol or drugs, or receive prior blood transfusions. No known allergies were reported.

Although she was stable and displayed minimal symptoms, her neurologist was nevertheless consulted regarding her anesthetic management due to the inherent risks attributed to the patient’s condition. Following the consultation, the anesthesiology team opted for general anesthesia. 

Her preoperative examination revealed a blood pressure of 104/76 mmHg, a heart rate of 81 beats per minute, and an oxygen saturation of 98% on room air. She was alert and oriented to person, place, and time. Cerebellar ataxia and dysarthria were also noted. Cardiac auscultation revealed a regular rate and rhythm with normal S1 and S2 heart sounds, and her lungs were clear bilaterally to auscultation. Her medication regimen included 1.25 mg weekly of ergocalciferol and ferrous sulfate, as needed. Laboratory results are shown in Table 2. Table 1 shows the clinical features of Alexander disease subtypes.

**Table 1:** Clinical features of Alexander disease subtypes.

Subtype	Age of Onset	Clinical Features	Neuroimaging Findings	Prognosis
Type I (Infantile)	<2 years	Macrocephaly, developmental delay, seizures, spasticity, failure to thrive	Frontal white matter abnormalities, contrast enhancement, ventricular enlargement	Rapidly progressive; poor prognosis
Type II (Adult-onset)	Adolescence to adulthood	Bulbar dysfunction (dysarthria, dysphagia), spasticity, ataxia, autonomic symptoms	Brainstem and spinal cord atrophy; medullary involvement; fewer frontal findings	Slowly progressive; variable outcome
Juvenile/Intermediate	2–12 years	Mixed features: spasticity, ataxia, cognitive decline, seizures	Variable — may have features of both Type I and Type II	Intermediate prognosis

**Table 2:** Pre-operative laboratory results. SGPT: Serum glutamic-pyruvic transaminase, SGOT: Serum glutamic-oxaloacetic transaminase

Complete Blood Panel (CBC)		
	*Results*	*Normal Range*
White Blood Count (X 10^3/uL)	10.54	3.98-10.04
Red Blood Count (X 10^3/uL)	4.95	3.93-5.22
Hemoglobin (gm/dL)	9.5	11.2-15.1
Platelets (X 10^3/uL)	218.00	163.00-369.00
Coagulation Panel		
	*Results*	*Normal Range*
Prothrombin Time (seconds)	10.50	9.00-11.50
Partial Thromboplastin Time (seconds)	28.30	22.20-34.00
International Normalized Ratio (INR)	0.99	0.90-1.10
Complete Metabolic Panel (CMP)		
	*Results*	*Normal Range*
Sodium (mEq/L)	137.90	135.00-145.00
Potassium (mEq/L)	4.40	3.30-5.10
Chloride (mEq/L)	105.00	98.00-107.00
Carbon Dioxide, Total (mEq/L)	26.00	25.00-30.00
Blood Urea Nitrogen (mg/dL)	12.00	7.00-20.00
Creatinine (mg/dL)	0.50	0.50-1.50
Glucose (mg/dL)	90.00	70.00-99.00
Alkaline Phosphatase (u/L)	56.00	38.00-126.00
SGPT (ALT) (u/L)	11.00	0.00-35.00
SGOT (AST) (u/L)	17.00	15.00-46.00
Albumin (g/dL)	3.90	3.50-5.00
Calcium (mg/dL)	9.10	8.40-10.20

The anesthetic plan for the procedure included preoperative administration of intravenous (IV) midazolam (5 mg), followed by induction of general anesthesia with propofol (120 mg, IV) and rocuronium (30 mg, IV). Given the uncertain but plausible risk of silent aspiration and the absence of definitive guidelines for extended NPO status in this population, our team opted for a modified rapid sequence induction strategy to mitigate peri-induction aspiration risk. Rocuronium was selected over succinylcholine due to both its availability and a more favorable side-effect profile, particularly in patients with neuromuscular vulnerability. Cricoid pressure was applied, and preoxygenation was achieved via controlled ventilation using low tidal volumes, aiming to shorten the apneic period and minimize the likelihood of regurgitation. Endotracheal intubation was performed using video-assisted laryngoscopy (McGrath, MAC 3) with a 7.0 mm endotracheal tube secured at 21 cm at the right labial commissure. Adequate positioning was confirmed by capnography and auscultation of bilateral breath sounds via stethoscope. Mechanical ventilation was initiated with a tidal volume of 425 mL and a respiratory rate of 10 breaths per minute. Anesthesia was maintained with inhaled sevoflurane at 2%, which was later reduced to 0.6% after the administration of 50 mcg of fentanyl due to a mild elevation in blood pressure. 

Throughout the procedure, her blood pressure ranged from 130/80 mmHg to 100/60 mmHg, her heart rate ranged between 65 and 95 bpm, and her oxygen saturation remained consistent at 100%. End-tidal CO₂ levels ranged from 34 to 45 mmHg. After an adequate train-of-four response of 4/4, sugammadex (200 mg, IV) was administered for the reversal of neuromuscular blockade. In addition, flumazenil (0.2 mg, IV) was given to reverse the effects of midazolam. The patient returned to a spontaneous ventilatory pattern with adequate tidal volumes and gas exchange. 

The procedure lasted 50 minutes and was completed successfully, without complications. The patient was extubated in the operating room and displayed adequate protective airway reflexes. She was transferred to the post-anesthesia care unit (PACU) with stable vital signs: blood pressure of 111/65 mmHg, heart rate of 69 bpm, respiratory rate of 16 breaths per minute, and an oxygen saturation of 100%. The patient did not require additional medications for postoperative pain management. She displayed consistent and stable vital signs after the procedure, with no remarkable events, and was discharged by the Obstetrics and Gynecology service later that evening. 

## Discussion

We present the successful management of a patient with Alexander’s disease type II undergoing laparoscopic BTS and IUD removal under general anesthesia. Although general anesthesia has been previously used in patients with Alexander's disease undergoing magnetic resonance imaging (MRI), lumbar punctures, gastrostomy tube placement, ventricular shunt placement, and scoliosis correction [4], to our knowledge, this is the first reported case describing its application in a gynecological procedure.

Patients with Alexander’s disease present significant anesthetic challenges during gynecologic procedures such as IUD removal and laparoscopic BTS. When considering anesthetic options for these patients, general anesthesia is a better option than neuraxial anesthesia due to the physiological demands of pneumoperitoneum, Trendelenburg positioning, and the patient’s underlying central nervous system involvement. The pneumoperitoneum increases intra-abdominal pressure and reduces venous return and cardiac output, while the Trendelenburg position elevates intracranial pressure and reduces pulmonary compliance. These effects are particularly concerning in Alexander’s disease patients, where autonomic dysregulation may blunt compensatory cardiovascular responses. The sympathetic blockade caused by neuraxial anesthesia can exacerbate these issues, leading to pronounced hypotension. Moreover, neuraxial anesthesia may not adequately address the visceral discomfort associated with peritoneal insufflation, even when extended to the T4-T6 dermatomes, making it a suboptimal choice for these procedures. In contrast, general anesthesia provides better hemodynamic control, along with the added benefits of airway protection, controlled ventilation, and profound neuromuscular relaxation. These factors contribute to improved hemodynamic conditions and reduce the risk of aspiration and respiratory compromise, making general anesthesia a safer and more reliable option for this patient population. Considering these factors, general anesthesia was selected as the most appropriate anesthetic approach for this patient.

Managing difficult airways in patients with leukodystrophies presents unique challenges, primarily due to bulbar dysfunction and abnormal muscle tone, both of which heighten the risk of aspiration [4]. Hasegawa et al. demonstrated that a combination of fiberoptic intubation and video laryngoscopy can effectively address this issue by facilitating airway control while preserving spontaneous ventilation [5]. Moreover, the use of remimazolam and remifentanil has been reported to further reduce aspiration risk in these patients. Their short half-lives and rapid reversibility enable more precise management during the emergence from anesthesia [6,7]. Despite the advantages of these new anesthetic agents, some patients experience delayed emergence following the administration of reversal agents, even though they ultimately recover from anesthesia without postoperative complications [8]. In our case, although the anesthesiology team anticipated difficult airway management due to the possibility of aspiration, we used midazolam and fentanyl because short-acting medications were unavailable. Although our patient did not develop this complication during surgery, we remained vigilant for its potential emergence in the postoperative period. In anticipation, we adhered to the American Society of Anesthesiologists (ASA) guidelines for patients with compromised airway reflexes, which recommend extended observation with continuous pulse oximetry and readiness for reintubation if necessary. Discharge was only considered after confirming stable respiratory function, intact protective airway reflexes, and adequate ventilation [8]. 

Another critical consideration in Alexander’s disease patients is autonomic instability, which can lead to labile blood pressure, bradycardia, or exaggerated hemodynamic responses during surgical stimulation [1,4]. These patients may also be at risk for autonomic dysreflexia, particularly in the setting of pelvic procedures. In gynecological surgery, such reflexes could be triggered by uterine manipulation or peritoneal insufflation. In our case, the patient’s intraoperative hemodynamics remained stable, but continuous monitoring was crucial given the theoretical risk of exaggerated reflex responses.

This case illustrates the use of commonly available anesthetic agents as an alternative approach in the context of national shortages and cost-related constraints affecting access to remifentanil and remimazolam. In the United States, remifentanil is a widely used short-acting synthetic opioid analgesic, primarily employed in anesthesia and critical care settings [9]. Remimazolam, on the other hand, is a novel benzodiazepine recently approved for use in procedural sedation and general anesthesia [10]. However, both drugs are associated with higher costs [11], and their availability can be inconsistent across hospitals [12]. Like many public and non-profit hospitals, our institution faces limited access to resources, which hinders its ability to acquire these more expensive medications.

## Conclusions

This case highlights the successful anesthetic management of a patient with symptomatic adult-onset Alexander’s disease undergoing laparoscopic gynecological surgery under general anesthesia. Key perioperative concerns, including aspiration risk due to bulbar dysfunction, potential altered responses to neuromuscular blocking agents from spasticity, and autonomic instability, were anticipated and carefully addressed through a modified rapid sequence induction, neuromuscular monitoring with sugammadex reversal, and continuous hemodynamic surveillance. The choice of general anesthesia was justified by the physiological demands of laparoscopy, including pneumoperitoneum and Trendelenburg positioning, which would have posed greater challenges under neuraxial techniques. Despite resource limitations that precluded the use of short-acting agents like remimazolam or remifentanil, this case demonstrates that with individualized planning and vigilance, standard anesthetic agents can be used safely and effectively. By detailing specific anesthetic considerations and mitigation strategies, this report contributes valuable insight into the perioperative care of patients with this rare neurodegenerative condition and may serve as a reference for future cases with greater neurologic severity.
